# Supplementary material for: Enhancing cross-protection against influenza by heterologous sequential immunization with mRNA LNP and protein nanoparticle vaccines
Source: Nat Commun. 2024 Jul 10;15:5800. doi: 10.1038/s41467-024-50087-5 (PMC11237032; doi:10.1038/s41467-024-50087-5)
Supplement: Supplementary file 1 — Supplementary Information [file 41467_2024_50087_MOESM1_ESM.pdf]

Supplementary Information

Enhancing Cross-Protection against Influenza by Heterologous Sequential Immunization with mRNA LNP and Protein Nanoparticles

Chunhong Dong[1], Wandi Zhu[1], Lai Wei[1], Joo Kyung Kim[1], Yao Ma[1], Sang-Moo Kang[1], and Bao-Zhong Wang[1]*

[1] Center for Inflammation, Immunity & Infection, Georgia State University Institute for Biomedical Sciences, 100 Piedmont Ave SE, Atlanta, Georgia 30303, USA

*Bao-Zhong Wang, Email: bwang23@gsu.edu

Contents:

Supplementary Text

Supplementary Fig. 1 to Fig. 9

Supplementary Text

Aichi HA coding sequence

ATGAAGACCATCATTGCTTTGAGCTACATTTTCTGTCTGGCTCTCGGCCAAGACCTTC
CAGGAAATGACAACAGCACAGCAACGCTGTGCCTGGGACATCATGCGGTGCCAAAC
GGAACACTAGTGAAAACAATCACAGATGATCAGATTGAAGTGACTAATGCTACTGA
GCTAGTTCAGAGCTCCTCAACGGGGAAAATATGCAACAATCCTCATCGAATCCTTGA
TGGAATAGACTGCACACTGATAGATGCTCTATTGGGGGACCCTCATTGTGATGTTTT
TCAAAATGAGACATGGGACCTTTTCGTTGAACGCAGCAAAGCTTTCAGCAACTGTTA
CCCTTATGATGTGCCAGATTATGCCTCCCTTAGGTCACTAGTTGCCTCGTCAGGCACT
CTGGAGTTTATCACTGAGGGTTTCACTTGGACTGGGGTCACTCAGAATGGGGGAAGC
AATGCTTGCAAAAGGGGACCTGGTAGCGGTTTTTTCAGTAGACTGAACTGGTTGAcC
AAATCAGGAAGCACATATCCAGTGCTGAACGTGACTATGCCAAACAATGACAATTT
TGACAAACTATACATTTGGGGGGTTCACCACCCGAGCACGAACCAAGAACAAACCA
GCCTGTATGTTCAAGCATCAGGGAGAGTCACAGTCTCTACCAGGAGAAGCCAGCAA
ACTATAATCCCGAATATCGGGTCCAGACCCTGGGTAAGGGGTCTGTCTAGTAGAATA
AGCATCTATTGGACAATAGTTAAGCCGGGAGACGTACTGGTAATTAATAGTAATGG
GAACCTAATCGCTCCTCGGGGTTATTTCAAAATGCGCACTGGGAAAAGCTCAATAAT
GAGGTCAGATGCACCTATTGATACCTGTATTTCTGAATGCATCACTCCAAATGGAAG
CATTCCCAATGACAAGCCCTTTCAAAACGTAAACAAGATCACATATGGAGCATGCC
CCAAGTATGTTAAGCAAAACACCCTGAAGTTGGCAACAGGGATGCGGAATGTACCA
GAGAAACAAACTAGAGGCCTATTCGGCGCAATAGCAGGTTTCATAGAAAATGGTTG
GGAGGGAATGATAGACGGTTGGTACGGTTTCAGGCATCAAAATTCTGAGGGCACAG
GACAAGCAGCAGATCTTAAAAGCACTCAAGCAGCCATCGACCAAATCAATGGGAAA
TTGAACAGGGTAATCGAGAAGACGAACGAGAAATTCCATCAAATCGAAAAGGAATT
CTCAGAAGTAGAAGGGAGAATTCAGGACCTCGAGAAATACGTTGAAGACACTAAAA
TAGATCTCTGGTCTTACAATGCGGAGCTTCTTGTCGCTCTGGAGAATCAACATACAA
TTGACCTGACTGACTCGGAAATGAACAAGCTGTTTGAAAAAACAAGGAGGCAACTG
AGGGAAAATGCTGAAGACATGGGCAATGGTTGCTTCAAAATATACCACAAATGTGA
CAACGCTTGCATAGAGTCAATCAGAAATGGGACTTATGACCATGATGTATACAGAG
ACGAAGCATTAAACAACCGGTTTCAGATCAAAGGTGTTGAACTGAAGTCTGGATAC
AAAGACTGGATCCTGTGGATTTCCTTTGCCATATCATGCTTTTTGCTTTGTGTTGTTTT
GCTGGGGTTCATCATGTGGGCCTGCCAGAGAGGCAACATTAGGTGCAACATTTGCAT
TTAG

Supplementary Figures

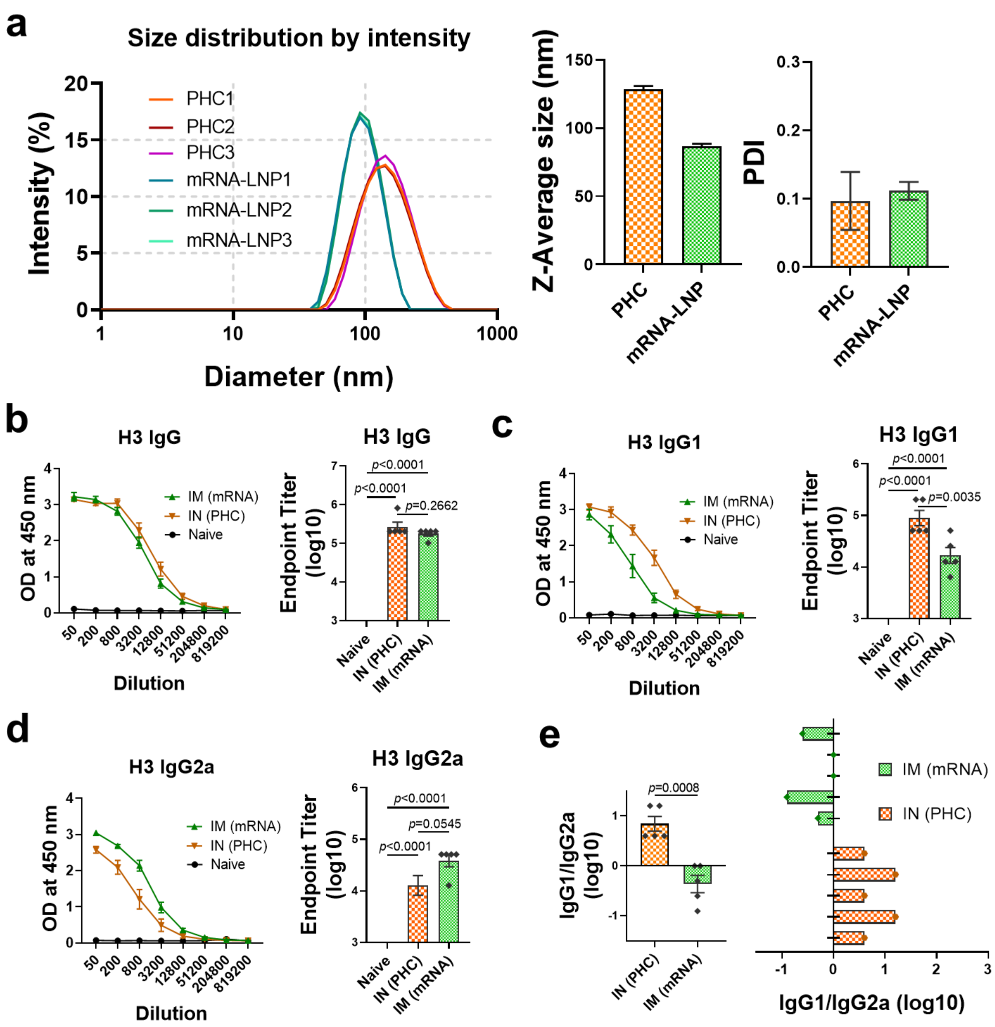

Supplementary Fig. 1. Characterizations of the IM mRNA and IN PHC vaccines. (**a**) Sizes and polydispersity index (PDI) of the mRNA-LNP and PHC nanoparticles determined by dynamic light scattering (*n*=3 samples). (**b-d**) Serum antibody responses post-one-dose IM (mRNA) or IN (PHC) immunization, respectively. Serum antigen (H3)-specific IgG (**b**), IgG1 (**c**), and IgG2a (**d**) antibody levels 3 weeks post-vaccination. (**e**) Serum IgG1/IgG2a (log10) values. The right panel depicts the results of individual mice. The sample size (*n*) was 5 for (**b-e**). Data are presented as mean ± SEM. Statistical significance was analyzed by one-way ANOVA with Turkey's multiple comparison tests (**b-d**) or unpaired two-tailed Student's *t*-test (**e**). Source data are provided as a Source Data file.

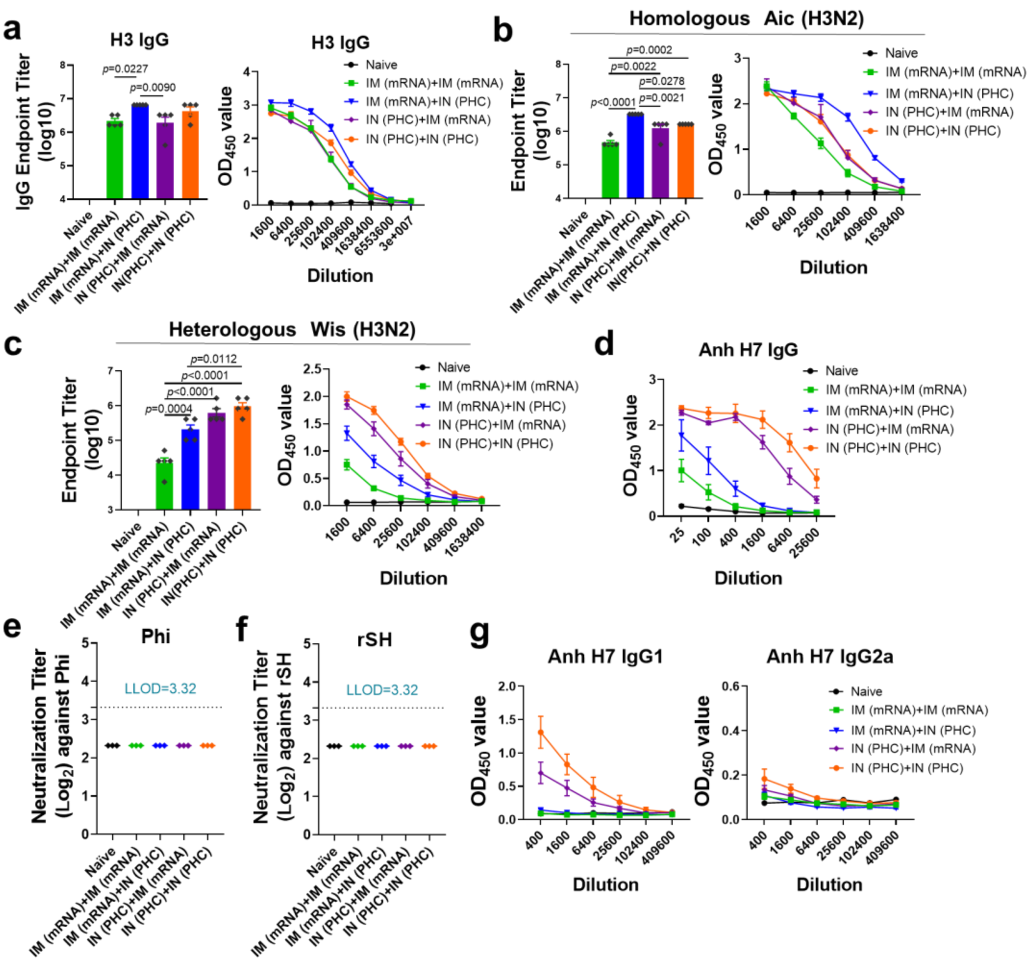

Supplementary Fig. 2. **Serum antibody cross-reactivity.** Immune sera were collected three weeks post-boost immunization. (**a-b**) IgG levels against H3 and homologous Aic virus. (**c-d**) Cross-reactive IgG antibodies against heterologous Wis and heterosubtypic Anh H7, respectively. (**e-f**) Serum neutralization activity against Phi and rSH viruses, respectively. (**g**) Anh H7-specific IgG1 and IgG2a antibody levels. Data are presented as mean ± SEM. The sample size *n* was 5 for (a-d, g) and 3 for (e-f). Statistical significance was analyzed by one-way ANOVA followed by Turkey's multiple comparison tests. Source data are provided as a Source Data file.

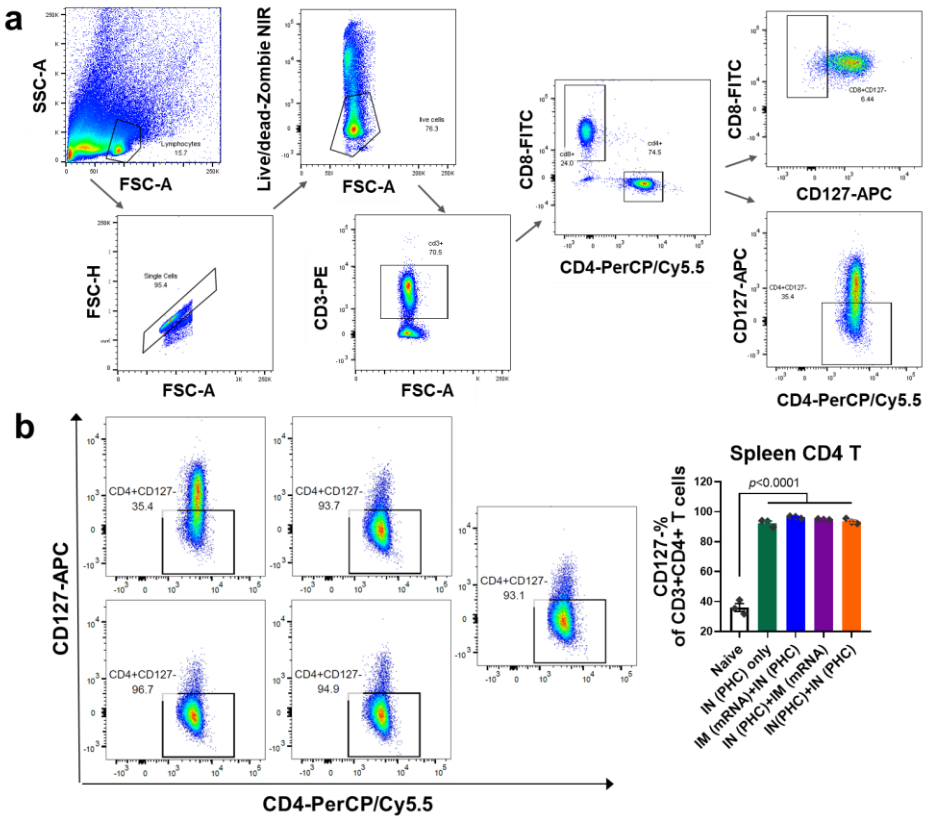

Supplementary Fig. 3. **CD127 expression on ex-vivo cultured mouse splenic T cells.** (**a**) The gating strategy of CD127 expression on mouse splenic CD4 and CD8 T cells after a 2-day antigen-restimulation. (**b**) CD127 expression on splenic CD4 T cells (*n*=3 samples). Spleens were collected 5 weeks post-boosting immunization. Data are presented as mean ± SEM. Statistical significance was analyzed by one-way ANOVA followed by Turkey's multiple comparison tests. Source data are provided as a Source Data file.

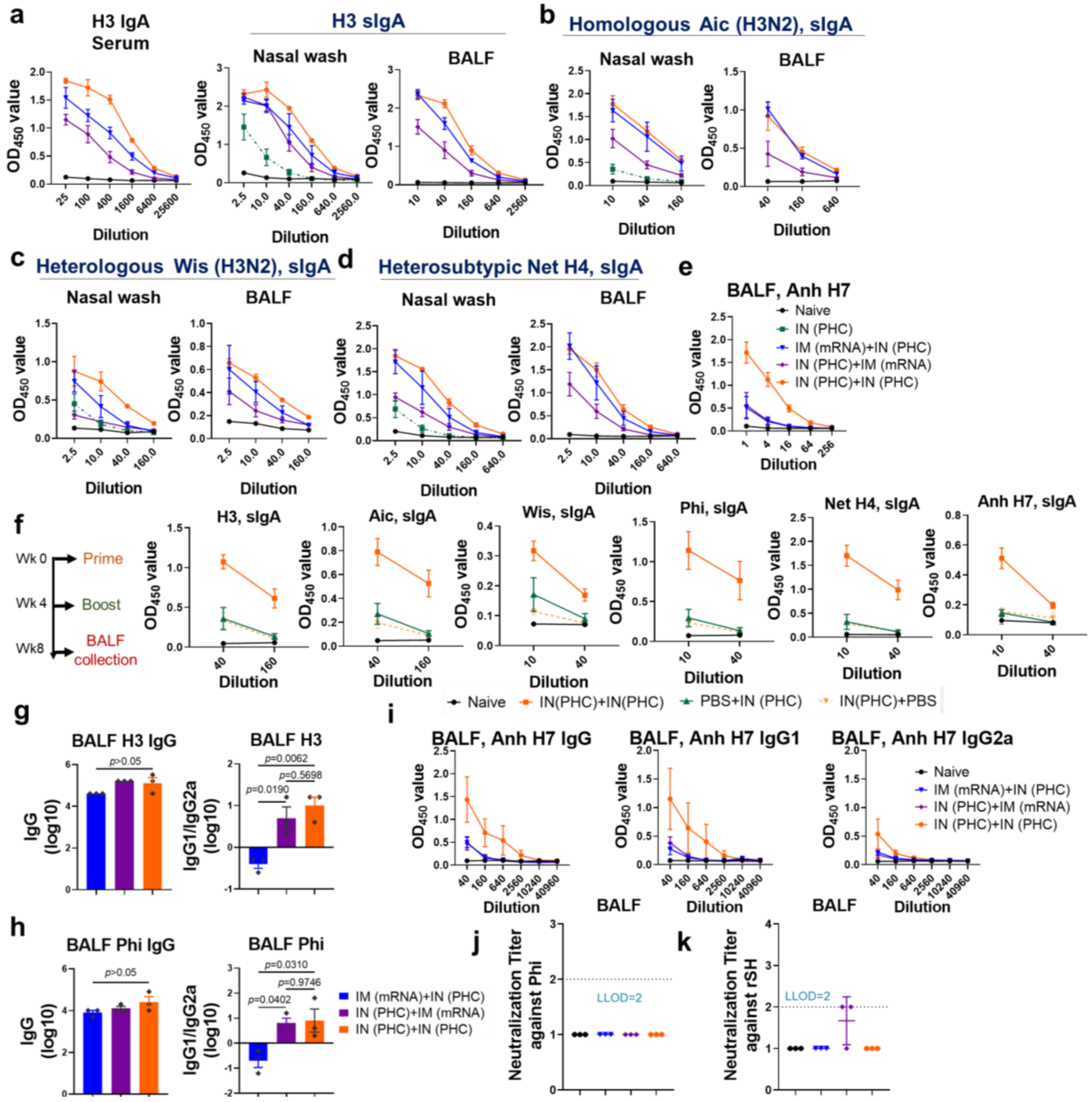

Supplementary Fig. 4. **Serum IgA and mucosal sIgA levels.** (**a**) H3-specific serum IgA levels and mucosal sIgA in nasal washes and BALF, respectively. (**b-e**) Mucosal sIgA cross-reactivity to Aichi, Wis, Net H4, and Anh H7 in nasal washes and BALF, respectively. Samples were collected 5 weeks post-boosting immunization. (**f**) A separate experiment to study the cross-reactive sIgA production in mouse BALF post-PHC vaccination. We immunized the mice with one or two doses of PHC at the indicated time points and determined the sIgA antibody levels against H3, Aic, Wis, Phi, Net H4, and Anh H7 by ELISA at week 8. (**g-h**) H3- and Phi-specific IgG levels and IgG1/IgG2a ratios in BALF. (**i**) Anh H7-specific IgG, IgG1, and IgG2a levels in BALF. (**j-k**) Neutralization activity against Phi and SH in BALF, respectively. Data are presented as mean ± SEM. The sample size *n*=5 for immune sera, and *n*=3 for BALF in immunized mice. Statistical significance was analyzed by one-way ANOVA followed by Turkey's multiple comparison tests. Source data are provided as a Source Data file.

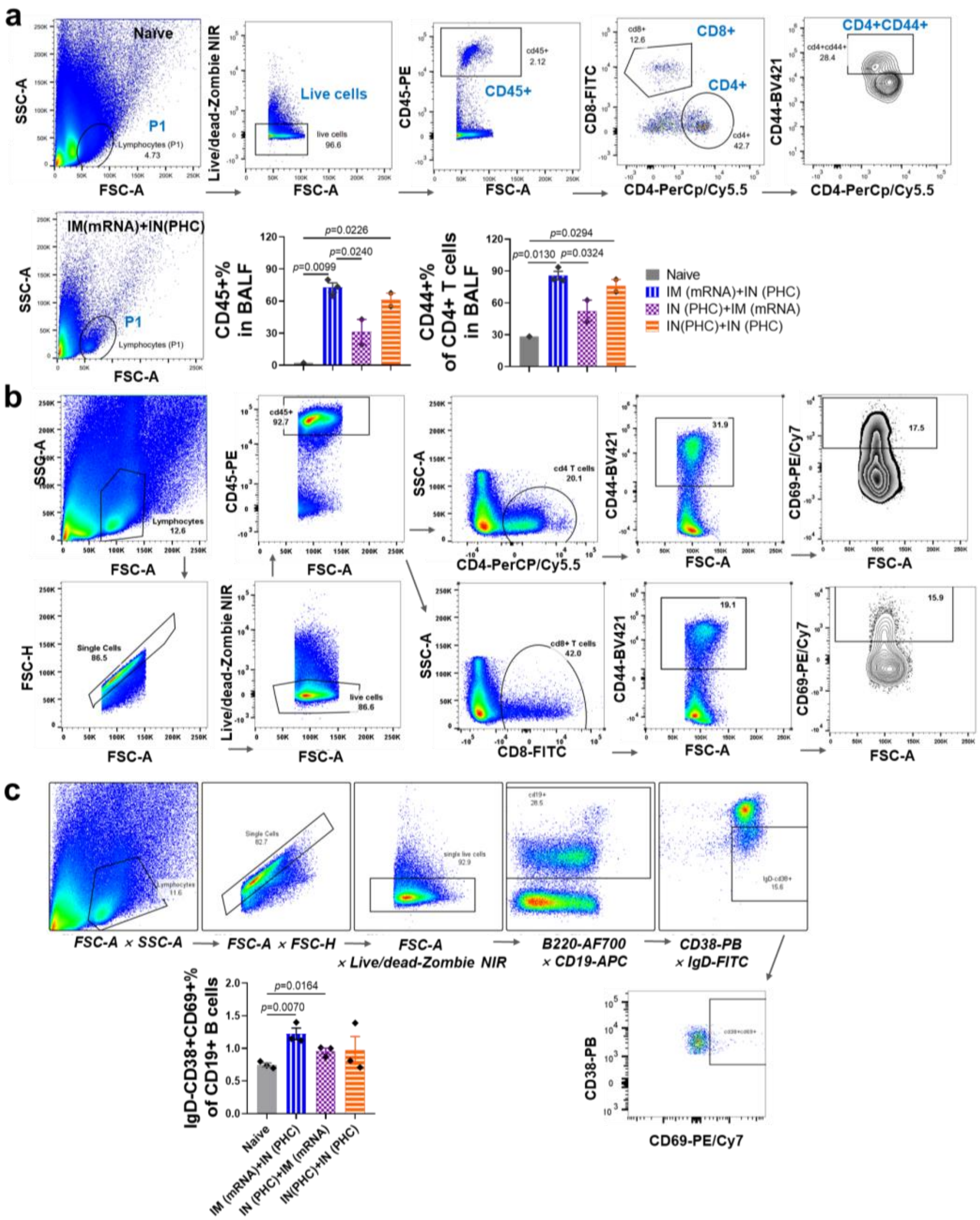

Supplementary Fig. 5. Flow cytometry analysis of BALF and lung cell populations. (**a**) The gating strategy and frequencies of CD45+ lymphocytes and CD4+CD44+ cells in BALF. Pooled cells were used for the naïve group. The sample size *n*=1/3/2/2 for specified groups depending on the cell abundance. (**b**) Gating strategies for lung CD4+CD44+CD69+ and CD8+CD44+CD9+ T cells. (**c**) Gating strategy and CD19+IgD-CD38+CD69+ B_{RM} frequencies in lung CD19+ B cells (*n*=3 samples). BALF and lung tissues were collected 5 weeks post-boosting immunization. Data are presented as mean ± SEM. Statistical significance was analyzed by one-way ANOVA followed by Dunnett's multiple comparison tests (**a**) or unpaired two-tailed Student's *t*-test (**c**). Source data are provided as a Source Data file.

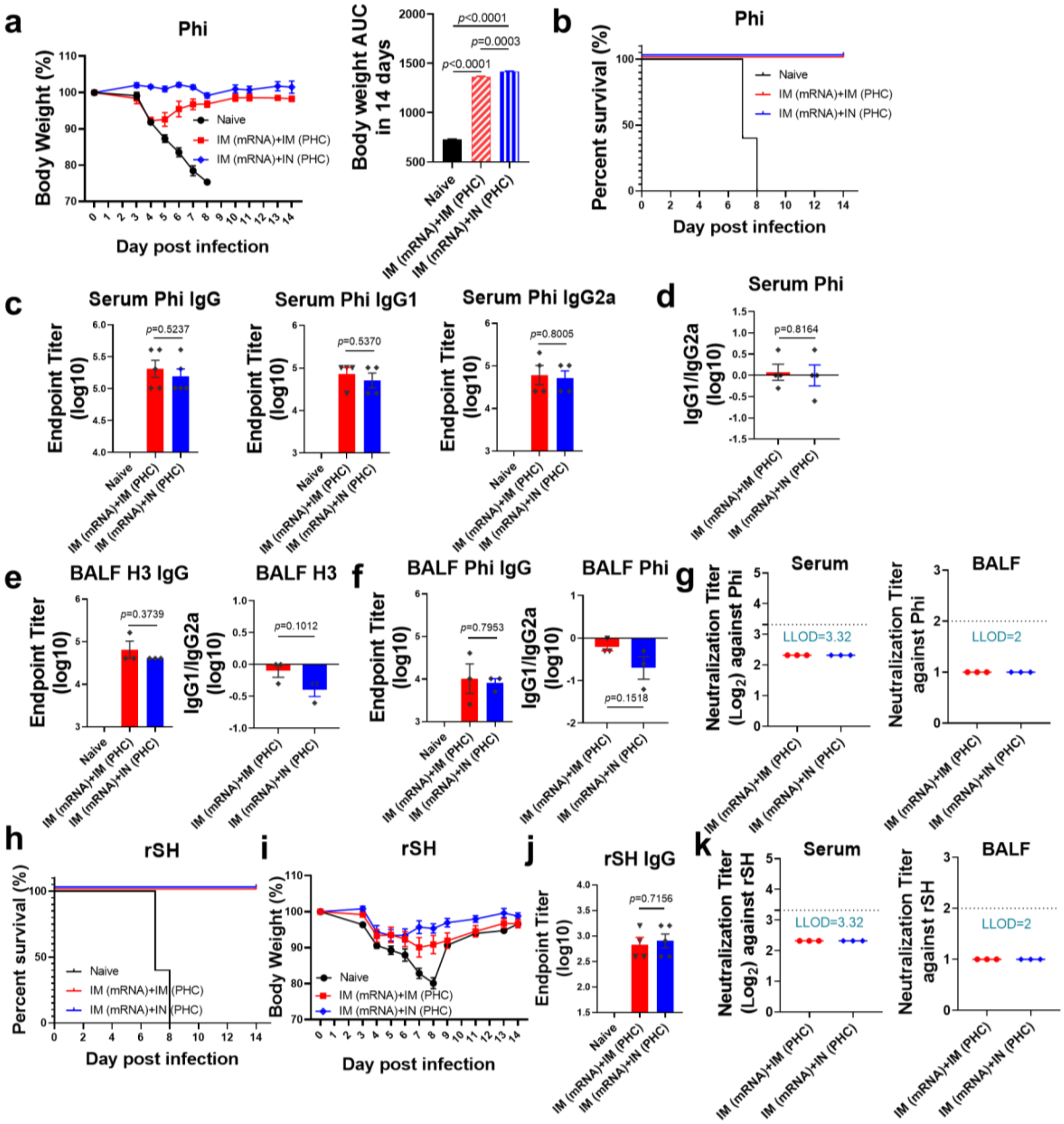

Supplementary Fig. 6. Comparison of the cross-protection efficacies between the IM (mRNA)+IN (PHC) and IM (mRNA)+IM (PHC) groups. (**a**) Mouse body weight changes and the 14-day bodyweight AUC post-challenge with 2×LD_{50} heterologous Phi. (**b**) Comparison of mouse survival rates post-Phi challenge. (**c-d**) Phi-specific IgG, IgG1, and IgG2a antibody levels and IgG1/IgG2a ratios in immune sera collected 3 weeks post-boosting immunization. (**e-f**) H3- and Phi-specific IgG levels and IgG1/IgG2a ratios in BALF. (**g**) Neutralizing activity against Phi in immune sera and BALF post-boosting immunization. (**h**) Comparison of mouse survival rates post-heterosubtypic rSH challenge. (**i**) Mouse body weight changes post-rSH (3×LD_{50}) challenge. (**j-k**) rSH-specific serum IgG antibody and neutralizing antibody levels in immune sera and BALF post-boosting immunization. LLOD, the lower limit of detection. Data are presented as mean ± SEM. The sample size *n*=5 for **a-b**, **c** (IgG), **h-i**, and **j** (IM (mRNA)+IM (PHC)); *n*=4 for **c** (IgG1 and IgG2a), **d**, and j (IM (mRNA)+IN (PHC)); *n*=3 for **e-g** and **k**. Statistical significance was analyzed by one-way ANOVA followed by Turkey's multiple comparison tests (**a**) or unpaired two-tailed Student's *t*-test (**c-j**). Source data are provided as a Source Data file.

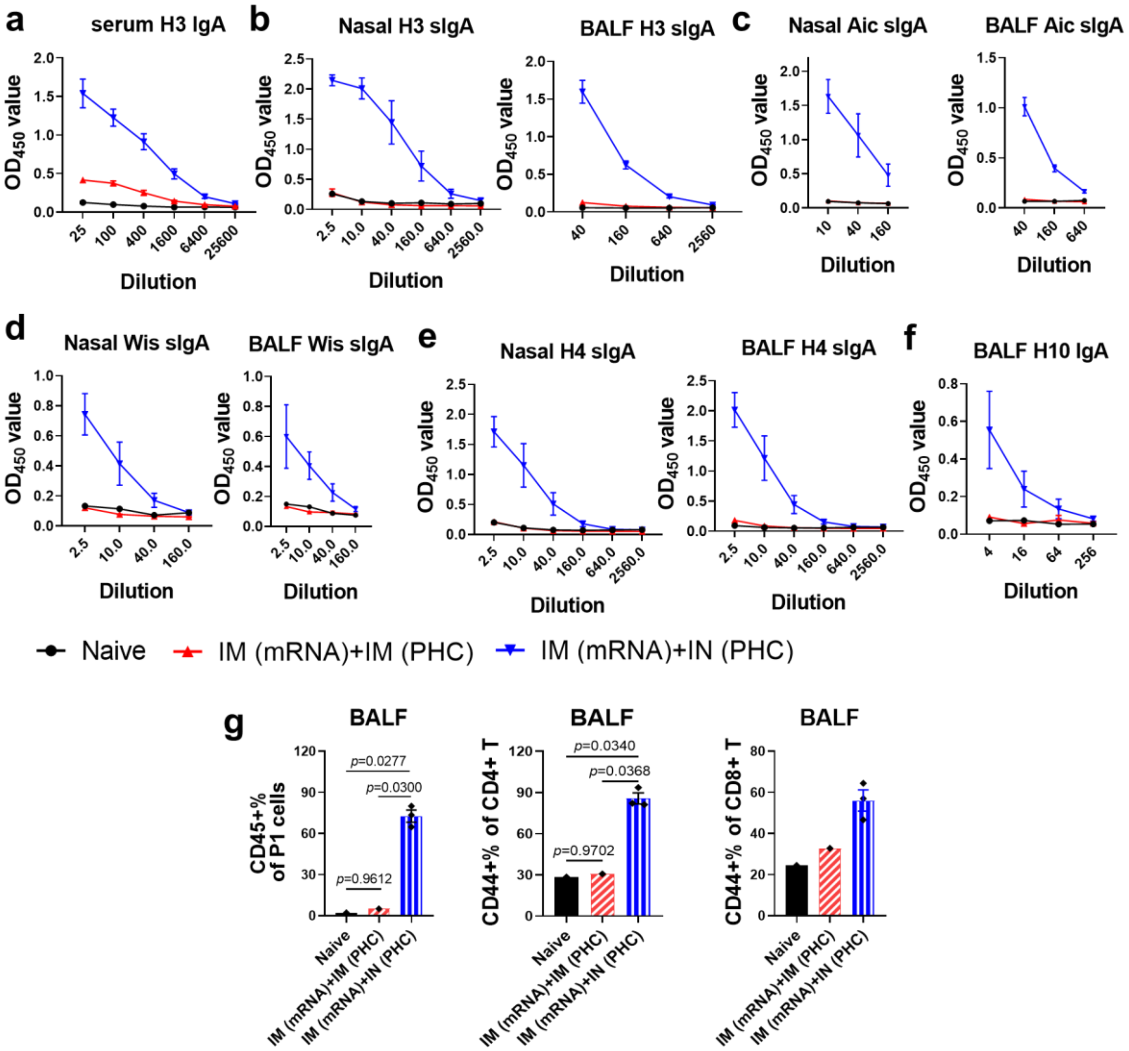

Supplementary Fig. 7. Comparison of the mucosal sIgA and cellular responses between the IM (mRNA)+IN (PHC) and IM (mRNA)+IM (PHC) groups. (**a**) Serum H3-specific IgA levels. The sample size *n*=3 for the naïve group and *n*=5 for other groups. (**b-f**) sIgA levels against H3, homologous Aic, heterologous Wis, and heterosubtypic Net H4 and Swe H10 in nasal washes and BALF, respectively. Immune sera and mucosal washes (nasal washes and BALF) were collected 3 and 5 weeks post-boosting immunization, respectively. The sample size *n*=1 for the naïve group and *n*=3 for other groups. (**g**) Comparison of airway CD45+, CD4+CD44+, and CD8+CD44+ lymphocytes. Pooled cells were used for the Naïve and IM (mRNA)+IM (PHC) groups (*n*=1). *n*=3 for the IM (mRNA)+IN (PHC) group. Data are presented as mean ± SEM. Statistical significance was analyzed by one-way ANOVA followed by Turkey's multiple comparison tests. Source data are provided as a Source Data file.

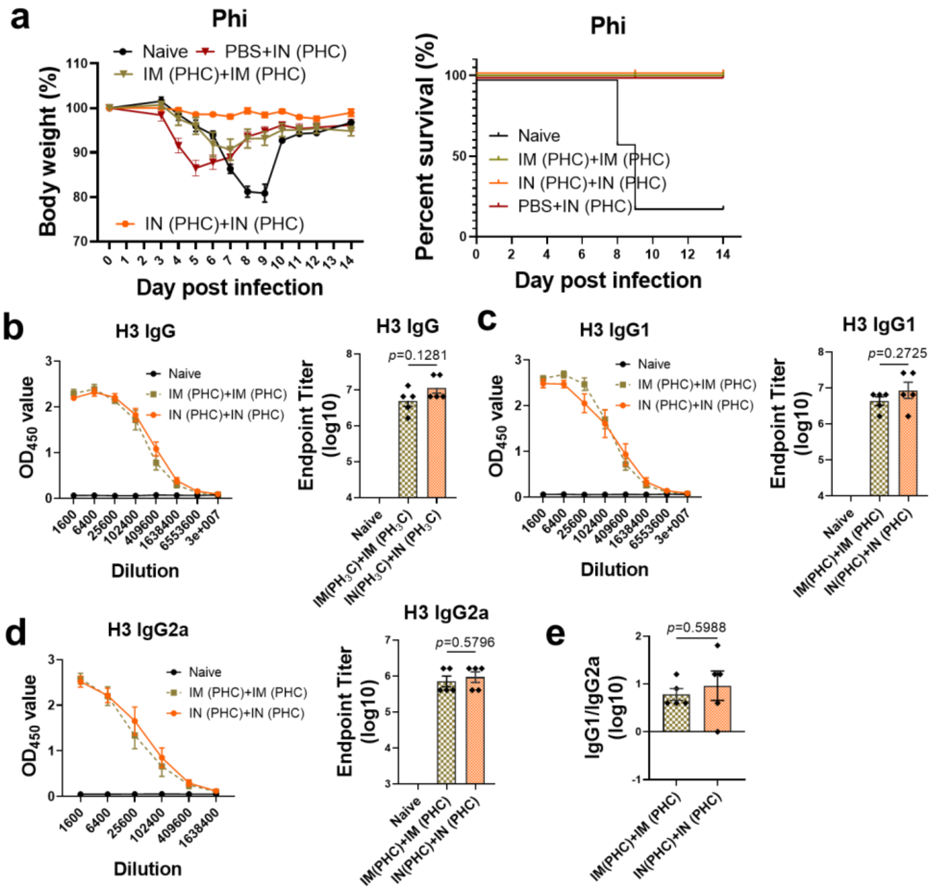

Supplementary Fig. 8. Comparison of the cross-protection efficacies between the IM (PHC)+IM (PHC) and IN (PHC)+IN (PHC) groups post-Phi (3×LD_{50}) challenge. (**a**) Mouse body weight changes and survival rates. (**b-d**) Antigen (H3)-specific IgG, IgG1, and IgG2a levels in immune sera collected 3 weeks post-boosting immunization. (**e**) Comparison of serum IgG1/IgG2a ratios. Data are presented as mean ± SEM (*n*=5 samples). Statistical significance was analyzed by unpaired two-tailed Student's-*t* test. Source data are provided as a Source Data file.

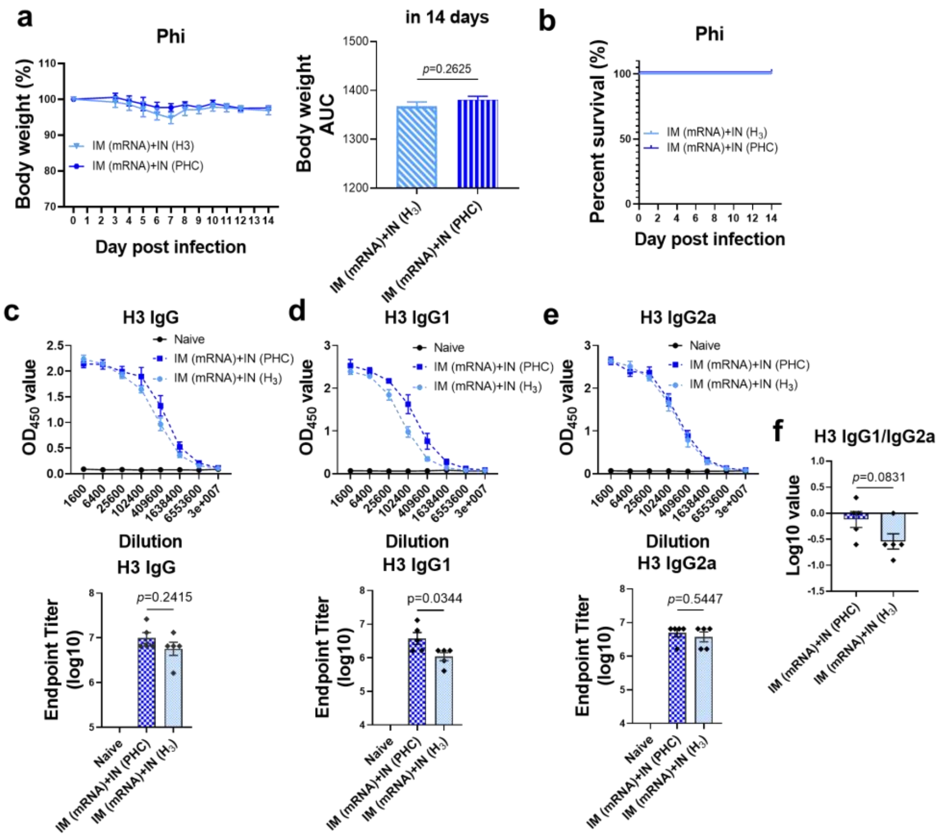

Supplementary Fig. 9. Comparison of the heterologous protection efficacies between IM (mRNA)+IN (H3) and IM (mRNA)+IN (PHC) groups against 3×LD_{50} Phi challenge. (**a**) Comparison of mouse body weight changes and 14-day bodyweight AUC post-Phi challenge. (**b**) Mouse survival rates post-challenge. (**c-f**) Antigen (H3)-specific IgG, IgG1, IgG2a antibody levels, and IgG1/IgG2a ratios (log10) in immune sera collected 3 weeks post-boosting immunization. Data are presented as mean ± SEM (n=5 samples). Statistical significance was analyzed by unpaired two-tailed Student's t-test. Source data are provided as a Source Data file.
