# Supplementary material for: Enhancing cross-protection against influenza by heterologous sequential immunization with mRNA LNP and protein nanoparticle vaccines
Source: Nat Commun. 2024 Jul 10;15:5800. doi: 10.1038/s41467-024-50087-5 (PMC11237032; doi:10.1038/s41467-024-50087-5)
Supplement: Supplementary file 3 — Reporting Summary [file 41467_2024_50087_MOESM3_ESM.pdf]

nature portfolio

Corresponding author(s): Bao-Zhong Wang

Last updated by author(s): Jun 24, 2024

Reporting Summary

Nature Portfolio wishes to improve the reproducibility of the work that we publish. This form provides structure for consistency and transparency in reporting. For further information on Nature Portfolio policies, see our Editorial Policies and the Editorial Policy Checklist.

Statistics

For all statistical analyses, confirm that the following items are present in the figure legend, table legend, main text, or Methods section.

n/a	Confirmed	
☐	☒	The exact sample size (*n*) for each experimental group/condition, given as a discrete number and unit of measurement
☐	☒	A statement on whether measurements were taken from distinct samples or whether the same sample was measured repeatedly
☐	☒	The statistical test(s) used AND whether they are one- or two-sided<br>*Only common tests should be described solely by name; describe more complex techniques in the Methods section.*
☒	☐	A description of all covariates tested
☐	☒	A description of any assumptions or corrections, such as tests of normality and adjustment for multiple comparisons
☐	☒	A full description of the statistical parameters including central tendency (e.g. means) or other basic estimates (e.g. regression coefficient) AND variation (e.g. standard deviation) or associated estimates of uncertainty (e.g. confidence intervals)
☐	☒	For null hypothesis testing, the test statistic (e.g. *F*, *t*, *r*) with confidence intervals, effect sizes, degrees of freedom and *P* value noted<br>*Give P values as exact values whenever suitable.*
☒	☐	For Bayesian analysis, information on the choice of priors and Markov chain Monte Carlo settings
☒	☐	For hierarchical and complex designs, identification of the appropriate level for tests and full reporting of outcomes
☒	☐	Estimates of effect sizes (e.g. Cohen's *d*, Pearson's *r*), indicating how they were calculated

Our web collection on statistics for biologists contains articles on many of the points above.

Software and code

Policy information about availability of computer code

Data collection	Flow cytometry data were collected using BD LSRFortessa Cell Analyzer (BD Biosciences).<br>Enzyme-linked immunosorbent assay data were collected using Biotek Epoch Microplate Reader (Agilent).<br>Enzyme-linked immunospot assay data were recorded with BIOSYS Bioreader-6000-E (BIOSYS).<br>Dynamic light scattering data were obtained using Malvern Zetasizer Nano ZS (Malvern Panalytical).
Data analysis	The data plots/graphs and all statistical analyses were created by GraphPad Prism v8.0. The mouse body weight AUC and its standard error for each group were calculated from the body weight curve by GraphPad Prism 8. FlowJo v.10 software was used for analyzing flow cytometry data.

For manuscripts utilizing custom algorithms or software that are central to the research but not yet described in published literature, software must be made available to editors and reviewers. We strongly encourage code deposition in a community repository (e.g. GitHub). See the Nature Portfolio guidelines for submitting code & software for further information.

Data

Policy information about availability of data

All manuscripts must include a data availability statement. This statement should provide the following information, where applicable:

- Accession codes, unique identifiers, or web links for publicly available datasets
- A description of any restrictions on data availability
- For clinical datasets or third party data, please ensure that the statement adheres to our policy

All data that support the findings of this study are available with the manuscript, and a source data file is supplied.

Research involving human participants, their data, or biological material

Policy information about studies with human participants or human data. See also policy information about sex, gender (identity/presentation), and sexual orientation and race, ethnicity and racism.

Reporting on sex and gender	N/A
Reporting on race, ethnicity, or other socially relevant groupings	N/A
Population characteristics	N/A
Recruitment	N/A
Ethics oversight	N/A

Note that full information on the approval of the study protocol must also be provided in the manuscript.

Field-specific reporting

Please select the one below that is the best fit for your research. If you are not sure, read the appropriate sections before making your selection.

☒ Life sciences ☐ Behavioural & social sciences ☐ Ecological, evolutionary & environmental sciences

For a reference copy of the document with all sections, see nature.com/documents/nr-reporting-summary-flat.pdf

Life sciences study design

All studies must disclose on these points even when the disclosure is negative.

Sample size	The sample size was determined by ethical considerations and availability, and based on existing data sets from previous experiments with similar readouts and expected statistical errors. All sample sizes are stated in the manuscript. n=5 per group for immune serum samples and mouse challenge studies; n=3 for the evaluation of cellular and mucosal responses.
Data exclusions	No data was excluded from the reported analyses.
Replication	Replication is described in the legends where applicable.
Randomization	Mice were randomly assigned to different groups.
Blinding	For all experiments, animal numbers were provided to investigators, and investigators were blinded to grouping names until after the generation of the raw data.

Reporting for specific materials, systems and methods

We require information from authors about some types of materials, experimental systems and methods used in many studies. Here, indicate whether each material, system or method listed is relevant to your study. If you are not sure if a list item applies to your research, read the appropriate section before selecting a response.

Materials & experimental systems

n/a	Involved in the study
	☒ Antibodies
	☒ Eukaryotic cell lines
☒	☐ Palaeontology and archaeology
	☒ Animals and other organisms
☒	☐ Clinical data
☒	☐ Dual use research of concern
☒	☐ Plants

Methods

n/a	Involved in the study
☒	☐ ChIP-seq
	☒ Flow cytometry
☒	☐ MRI-based neuroimaging

Antibodies

Antibodies used

HRP-conjugated goat anti-mouse IgG, SouthernBiotech, Cat: 1033-05, Lot: J3316-P623D
HRP-conjugated goat anti-mouse IgG1, SouthernBiotech, Cat: 1071-05, Lot: K1619-N733
HRP-conjugated goat anti-mouse IgG2a, SouthernBiotech, Cat: 1080-05, Lot: B4520-RC63B
HRP-conjugated goat anti-mouse IgA, SouthernBiotech, Cat:1040-05, Lot: J4416-M729
mouse anti-influenza A virus NP antibody, Clone: C43, abcam, Ab128193.
anti-mouse IL-4, Clone: 11B11, Biolegend, Cat: 504102
anti-mouse IL-2, Clone: JES6-1A12, Biolegend, Cat: 503704
anti-mouse IFN-γ, Clone: R4-6A2, BD Biosciences, Cat: 551216
biotin-conjugated anti-mouse IL-4, Clone: BVD6-24G2, Biolegend, Cat: 504202
biotin-conjugated anti-mouse IL-2, Clone: JES6-5H4, Biolegend, Cat: 503804
biotin-conjugated anti-mouse IFN-γ, Clone: XMG1.2, BD Biosciences, Cat: 554410
anti-mouse CD45-PE, Clone: 30-F11, Biolegend, Cat:103105
anti-mouse CD4-PerCP/Cyanine5.5, Clone: RM4-5, BD Pharmingen™, Cat:550954
anti-mouse CD8α-FITC, Clone: 53-6.7, Biolegend, Cat:100712
anti-mouse CD44-BV421, Clone: IM7, Biolegend, Cat:103040
anti-mouse CD69-PE/Cy7, Clone: H1.2F3, Biolegend, Cat:104511
anti-mouse CD3e-PE, Clone: 145-2c11, eBioscience, Cat:12-0031-82
anti-mouse CD127-APC, Clone: A7R34, Biolegend, Cat:135011
anti-mouse CD19-APC, Clone: 1D3, BD Pharmingen™, Cat:550992
anti-mouse CD45R/B220-AF700, Clone: RA3-6B2, Biolegend, Cat:103231
anti-mouse IgD-FITC, Clone: 11-26c.2a, Biolegend, Cat:405703
anti-mouse CD38-Pacific Blue, Clone: 90, Biolegend, Cat:102719
Zombie NIR Fixable Viability Kit, Biolegend, Cat:423105
CD16/32, Clone: 2.4G2, BD Pharmingen™, Cat:553142
Mouse anti-influenza A virus NP antibody, Clone: C43, Ab128193, abcam
For ELISA assay, the HRP-conjugated goat anti-mouse IgG, IgG1, IgG2a, and IgA antibodies were used at a dilution raning from 1:2000 to 1:4000; mouse anti-influenza A virus NP antibody was used at a dilution of 1:2000. For B cell Elispot assays, the HRP-conjugated goat anti-mouse IgG, IgG1, IgG2a, and IgA antibodies were used at a dilution of 1:1000. For T cell Elispot assays, the capture antibodies were used at a dilution of 1:250; the biotin-conjugated detection antibodies were used at a dilution of 1:500. For the FACS assays, the antibodies were used at a dilution of 1:150. For the NP-based ELISA assay in microneutralization assays, the anti-NP antibody was used at a dilution of 1:2000.

Validation

All the antibodies were freshly purchased from the companies that provided certificates of validation analysis.
HRP-conjugated goat anti-mouse IgG: https://www.southernbiotech.com/goat-anti-mouse-igg-fc-hrp-1033-05; Development of neutralizing monoclonal antibodies against the pandemic H1N1 virus (2009) using plasmid DNA immunogen. J Virol Methods. 2014;195:54-62.
HRP-conjugated goat anti-mouse IgG1: https://www.southernbiotech.com/goat-anti-mouse-igg1-hrp-1071-05; The IκB kinase inhibitor ACHP strongly attenuates TGFβ1-induced myofibroblast formation and collagen synthesis. J Cell Mol Med. 2015 Sep 4. doi: 10.1111/jcmm.12661.
HRP-conjugated goat anti-mouse IgG2a: https://www.southernbiotech.com/goat-anti-mouse-igg2a-human-ads-hrp-1080-05; Non-lethal viral challenge of influenza haemagglutinin and nucleoprotein DNA vaccinated mice results in reduced viral replication. Scand J Immunol. 2002;55:14-23.
HRP-conjugated goat anti-mouse IgA: https://www.southernbiotech.com/goat-anti-mouse-iga-hrp-1040-05; Partial IgA-deficiency with increased Th2-type cytokines in TGF-β1 knockout mice. J Immunol. 1999;163:1951-7.
mouse anti-influenza A virus NP antibody: https://www.abcam.com/products/primary-antibodies/influenza-a-virus-nucleoprotein-antibody-c43-ab128193.html; Liu HY et al. Single-virus tracking with quantum dots in live cells. Nat Protoc 18:458-489 (2023).
anti-mouse IL-4: https://www.biolegend.com/nl-nl/products/purified-anti-mouse-il-4-antibody-894
anti-mouse IL-2: https://www.biolegend.com/en-us/products/ultra-leaf-purified-anti-mouse-il-2-antibody-7749
anti-mouse IFN-γ: https://www.bdbiosciences.com/en-us/products/reagents/immunoassay-reagents/purified-rat-anti-mouse-ifn.551216
biotin-conjugated anti-mouse IL-4: https://www.biolegend.com/en-us/products/biotin-anti-mouse-il-4-antibody-925
biotin-conjugated anti-mouse IL-2: https://www.biolegend.com/en-us/products/biotin-anti-mouse-il-2-antibody-951
biotin-conjugated anti-mouse IFN-γ: https://www.bdbiosciences.com/en-us/products/reagents/immunoassay-reagents/elisa/biotin-rat-anti-mouse-ifn.554410

CD45-PE: https://www.biolegend.com/nl-nl/products/pe-anti-mouse-cd45-antibody-100
CD4-PerCP/Cyanine5.5: https://www.bdbiosciences.com/en-us/products/reagents/flow-cytometry-reagents/research-reagents/single-color-antibodies-ruo/percp-cy-5-5-rat-anti-mouse-cd4.550954
CD8α-FITC: https://www.biolegend.com/nl-nl/products/apc-anti-mouse-cd8a-antibody-150
CD44-BV421: https://www.biolegend.com/nl-nl/products/brilliant-violet-421-anti-mouse-human-cd44-antibody-7225
CD69-PE/Cy7: https://www.biolegend.com/nl-nl/products/pe-cyanine7-anti-mouse-cd69-antibody-3168
CD3e-PE: https://www.thermofisher.com/antibody/product/CD3e-Antibody-clone-145-2C11-Monoclonal/12-0031-82
CD127-APC: https://www.biolegend.com/nl-nl/products/apc-anti-mouse-cd127-il-7ralpha-antibody-6191
CD19-APC: https://www.bdbiosciences.com/en-us/products/reagents/flow-cytometry-reagents/research-reagents/single-color-antibodies-ruo/apc-rat-anti-mouse-cd19.550992
CD45R/B220-AF700: https://www.biolegend.com/nl-nl/products/alexa-fluor-700-anti-mouse-human-cd45r-b220-antibody-3408
IgD-FITC: https://www.biolegend.com/nl-nl/products/fitc-anti-mouse-igd-1378
CD38-Pacific Blue: https://www.biolegend.com/nl-nl/products/pacific-blue-anti-mouse-cd38-antibody-6652
Zombie NIR Fixable Viability Kit: https://www.biolegend.com/nl-nl/products/zombie-nir-fixable-viability-kit-8657
CD16/32: https://www.bdbiosciences.com/en-us/products/reagents/flow-cytometry-reagents/research-reagents/single-color-antibodies-ruo/purified-rat-anti-mouse-cd16-cd32-mouse-bd-fc-block.553142
Mouse anti-influenza A virus NP antibody, Clone: C43, Ab128193, abcam: https://www.abcam.com/products/primary-antibodies/influenza-a-virus-nucleoprotein-antibody-c43-ab128193.html

Eukaryotic cell lines

Policy information about cell lines and Sex and Gender in Research

Cell line source(s)	Spodoptera frugiperda 9 (Sf9, American Type Culture Collection (ATCC), CRL-1711) cells and Madin–Darby Canine Kidney cells (MDCK (NBL-2), ATCC CCL-34) were purchased from ATCC and maintained in the lab as recommended by the vendor. These cell lines have been used in numerous studies.
Authentication	No authentication has been used for this study.
Mycoplasma contamination	Cell lines used have not tested positive for mycoplasma contamination.
Commonly misidentified lines (See ICLAC register)	No commonly misidentified lines were used in this study.

Animals and other research organisms

Policy information about studies involving animals; ARRIVE guidelines recommended for reporting animal research, and Sex and Gender in Research

Laboratory animals	Female BALB/c mice (6-8 weeks old) were purchased from Envigo and housed at Georgia State University Animal Facility at 20-23 °C, 45-55% relative humidity with 12-hour light/dark cycles, free food, and water supplies.
Wild animals	No wild animals were used in this study.
Reporting on sex	Mice used in this study were female. The findings are supposed to apply to both sexes. Female mice were chosen because males are aggressive and often fight and get injured, which interferes with data collection.
Field-collected samples	No field collected samples were used in this study.
Ethics oversight	The entire study was approved by Georgia State University Institutional Animal Care and Use Committee (IACUC). All Balb/c mouse experiments were performed in strict compliance with the IACUC guidelines of Georgia State University under IACUC protocol A22029.

Note that full information on the approval of the study protocol must also be provided in the manuscript.

Plants

Seed stocks	N/A
Novel plant genotypes	N/A
Authentication	N/A

Flow Cytometry

Plots

Confirm that:

- [x] The axis labels state the marker and fluorochrome used (e.g. CD4-FITC).
- [x] The axis scales are clearly visible. Include numbers along axes only for bottom left plot of group (a 'group' is an analysis of identical markers).
- [x] All plots are contour plots with outliers or pseudocolor plots.
- [x] A numerical value for number of cells or percentage (with statistics) is provided.

Methodology

Sample preparation	Sample preparation details were described in the Methods section.
Instrument	BD LSRFortessa Cell Analyzer (BD Biosciences)
Software	FlowJo v.10 software
Cell population abundance	Abundant immune cells can be obtained from mouse spleens and lungs. The T cell numbers in the BALF, particularly in naïve or IM-immunized mice, are comparatively low. Therefore, we utilized pooled cells for specified groups when cell numbers were limited.
Gating strategy	For Lung/BALF T cell analysis, cells were gated for cells, singlets, live/dead, CD45+, CD4+ or CD8+, which were further gated for the specific populations. CD44+ cells were defined as antigen-experienced T cells. CD44+CD69+ T cells were defined as resident T cells. For spleen T cell analysis, cells were gated for cells, singlets, live/dead, CD3+, CD4+/CD8+, which were further gated for the specific populations. CD127 expression, as a selective characteristic marker for long-lived memory T cells was evaluated. For lung B cell analysis, cell were gated for cells, singlets, live/dead, CD19+, IgD-CD38+, and CD69+ to select the Brm cells.

- [x] Tick this box to confirm that a figure exemplifying the gating strategy is provided in the Supplementary Information.
